# The prevalence of HIV among adults with pulmonary TB at a population level in Zambia

**DOI:** 10.1186/s12879-017-2345-5

**Published:** 2017-03-29

**Authors:** Pascalina Chanda-Kapata, Nathan Kapata, Eveline Klinkenberg, Martin P. Grobusch, Frank Cobelens

**Affiliations:** 1 Department of Disease Surveillance and Research, Ministry of Health, Lusaka, Zambia; 2 Center of Tropical Medicine and Travel Medicine, Department of Infectious Diseases, Division of Internal Medicine, Academic Medical Center, University of Amsterdam, Amsterdam, the Netherlands; 3 KNCV Tuberculosis Foundation, The Hague, the Netherlands; 4 Department of Global Health, Academic Medical Centre, University of Amsterdam, Amsterdam Institute for Global Health and Development, Amsterdam, the Netherlands

**Keywords:** Tuberculosis, Population, HIV-TB co-morbidity

## Abstract

**Background:**

Tuberculosis and HIV co-infection is one of the main drivers of poor outcome for both diseases in Zambia. HIV infection has been found to predict TB infection/disease and TB has been reported as a major cause of death among individuals with HIV. Improving case detection of TB/HIV co-infection has the potential to lead to early treatment of both conditions and can impact positively on treatment outcomes. This study was conducted in order to determine the HIV prevalence among adults with tuberculosis in a national prevalence survey setting in Zambia, 2013–2014.

**Methods:**

A countrywide cross sectional survey was conducted in 2013/2014 using stratified cluster sampling, proportional to population size for rural and urban populations. Each of the 66 countrywide clusters represented one census supervisory area with cluster size averaging 825 individuals. Socio-demographic characteristics were collected during a household visit by trained survey staff. A standard symptom-screening questionnaire was administered to 46,099 eligible individuals across all clusters, followed by chest x-ray reading for all eligible. Those symptomatic or with x-ray abnormalities were confirmed or ruled out as TB case by either liquid culture or Xpert MTBRif performed at the three central reference laboratories. HIV testing was offered to all participants at the survey site following the national testing algorithm with rapid tests. The prevalence was expressed as the proportion of HIV among TB cases with 95% confidence limits.

**Results:**

A total of 265/6123 (4.3%) participants were confirmed of having tuberculosis. Thirty-six of 151 TB survey cases who accepted HIV testing were HIV-seropositive (23.8%; 95% CI 17.2–31.4). The mean age of the TB/HIV cases was 37.6 years (range 24–70). The majority of the TB/HIV cases had some chest x-ray abnormality (88.9%); were smear positive (50.0%), and/or had a positive culture result (94.4%). None of the 36 detected TB/HIV cases were already on TB treatment, and 5/36 (13.9%) had a previous history of TB treatment. The proportion of TB/HIV was higher in urban than in the rural clusters. The HIV status was unknown for 114/265 (43.0%) of the TB cases.

**Conclusions:**

The TB/HIV prevalence in the general population was found to be lower than what is routinely reported as incident TB/HIV cases at facility level. However; the TB/HIV co-infection was higher in areas with higher TB prevalence. Innovative and effective strategies for ensuring TB/HIV co-infected individuals are detected and treated early are required.

## Background

People living with TB are 26–31 times more likely to be co-infected with HIV, particularly in sub-Saharan Africa [1–3], with co-infection rates having been reported as high as 95% in Southern Africa [4]. The diagnosis of TB remains a challenge especially in areas where access to health services is limited and in view of HIV-positivity being associated with a high rate of extra-pulmonary TB, which offers formidable diagnostic challenges on its own [5].

In Zambia, the HIV prevalence among adults in 2014 was estimated to be 13%; with more cases in urban than rural areas, this representing a decline from the 16% prevalence reported in 2001 [6]. The prevalence of all forms of tuberculosis for all ages in 2013–2014 in Zambia was estimated to be 455/100,000 and bacteriologically confirmed TB among adults was 638/100,000 [7], underscoring the fact that Zambia has a high burden of both TB and HIV.

TB/HIV co-infection is one of the main drivers of poor outcome for both diseases and has been widely studied previously, including in Zambia [8, 9]. HIV infection has been found to predict TB infection/disease [1] and TB has been reported as a major cause of death among individuals with HIV [10–12]. Improving case detection of TB/HIV co-infection has the potential to lead to early treatment of both conditions and can impact positively on treatment outcomes [13].

In view of this, there is a clear need to better understand the HIV prevalence rate among TB patients and determine the co-infection rate at a population level. The Zambia national TB prevalence survey, the first to offer HIV testing to all participants provided an excellent opportunity to investigate this. This is the first study to document the national level prevalence of TB/HIV in a general population in Zambia.

## Methods

This was a cross sectional survey of the HIV status of the TB cases diagnosed during the national TB prevalence survey implemented by the Ministry of Health in Zambia. Nationwide data was collected among 66 clusters from September 2013 to July 2014. The sampling frame was nationwide clusters which were selected using random sampling proportional to size, stratified by rural and urban census supervisory areas (CSAs). The study participants were aged 15 years and above residing in the area. Survey participants were first screened for symptoms (chest pain or fever or cough lasting 2 weeks or more) and subsequently a chest x-ray was performed regardless of the screening result. Participants screening positive for symptoms or having an abnormal chest x-ray submitted a spot and morning sputum for mycobacterial (MTB) investigation at any of the three central reference laboratories. The details of the enrolment, screening and laboratory investigations are outlined elsewhere [7].

The TB cases were defined as cases having a positive *Mycobacterium tuberculosis* (MTB) result by liquid culture and or by the XpertMTB/RIF assay regardless of their TB treatment status at the time of the survey. The detailed methods for bacteriological confirmation of TB cases are outlined elsewhere [7]. Rapid HIV testing (with tiebreakers) was performed on site on an opt-out basis accompanied with pre and post-test counselling among consenting individuals using the national algorithm. The HIV testing procedure details were described earlier by Chanda-Kapata et al. [14].

Data on HIV status, TB status and relevant socio-demographic characteristics were extracted from the main survey database for all detected TB cases. The socio-demographic information collected included age, sex, location, education level, marital status and socio-economic status. The socio-economic status was measured using wealth indices as described earlier [14]. The symptom and chest x-ray screening outcome was also recorded; including TB treatment history for both participants and household members.

Data analysis was performed using STATA version 12. The prevalence of HIV among the TB cases was estimated as a proportion with 95% confidence limits. The difference in outcomes for binary variables was established using Pearson chi-square test while the Kruskall Wallis difference in proportions was used for multiple categorical variables.

## Ethical considerations

The study protocol was cleared by the University of Zambia Biomedical Research Ethics Committee (UNZABREC) No: 020–08-12. Authorisation to conduct the survey was sought in line with the existing national policies and guidelines at national, provincial and district levels. The Institutional Review Board (IRB) approved this consent procedure. All the consent or assent forms were recorded on standard forms which were developed for the study and these were filed in lockable cabinets at the end of each cluster operation and stored in central archives as per national requirements.

## Results

Overall, 46,099 individuals (84.1% of eligibles) participated in the TB survey; 6708 (14.6%) were eligible for sputum investigations and 265 (265/6123; 4.3%) bacteriologically confirmed TB cases were identified. Out of the 265 MTB cases, 151 (57.0%) agreed to be tested for HIV; 36 (23.8%; 95% CI 17.2–31.4) of whom were co-infected with HIV as shown in Table 1. The mean age was 37.6 (range 24–70) years. The proportion of individuals with TB/HIV co-infection was significantly higher among the urban (52.8%) versus rural (47.2%) residents (*p* = 0.001). There were no significant differences in the prevalence of TB/HIV co-infection by sex and education level. Participants aged 35–44 years were found to have the highest prevalence of TB/HIV (50%; *p* = 0.0001) compared to other age groups. More than two-thirds of the TB/HIV cases were currently married (63.9%; *p* < 0.05) and 50.0% were from the fourth wealth quintile (*p* < 0.05).

**Table 1:** Sociodemographic characteristics of the TB/HIV cases

Variable	N	% (95% CI)	*P*-value
Overall	36	23.8 (17.2–31.4)	
Location			
Rural	17	47.2 (30.4–64.5)	0.001^b^
Urban	19	52.8 (35.5–69.6)	
Sex			
Female	19	52.8 (35.5–69.6)	0.687^b^
Male	17	47.2 (30.4–64.5)	
Age group			
15–24	1	2.8 (0.0–14.5)	0.0001^c^
25–34	13	36.1 (20.8–53.8)	
35–44	18	50.0 (32.9–67.1)	
45–54	1	2.8 (0.0–14.5)	
55–64	2	5.6 (0.7–18.7)	
65+	1	2.8 (0.0–14.5)	
Education Level			
None	2	5.6 (0.7–18.7)	0.187^c^
Primary	19	52.8 (35.5–69.6)	
Secondary	13	36.0 (20.8–53.8)	
Tertiary	2	5.6 (0.7–18.7)	
Marital status			
Never married	3	8.3 (1.8–22.5)	0.003^c^
Currently married	23	63.9 (46.2–79.2)	
Divorced	9	25.0 (12.1–42.2)	
Widowed	1	2.8 (0.0–14.5)	
Wealth quintile^a^			
Lowest	3	10.7 (2.3–28.2)	0.0001^c^
Second lowest	1	3.6 (0.1–18.3)	
Middle	4	14.3 (4.0–32.7)	
Fourth	14	50.0 (30.6–69.4)	
Highest	6	21.4 (8.3–41.0)	

^a^The wealth quintile was unknown for 8 cases

^b^Pearson chi square test

^c^Kruskal-Wallis

The majority of the TB/HIV cases were from Copperbelt (*n* = 14; 39%) and Lusaka (*n* = 10; 28%) provinces respectively; 4 (11%) from Western province and two each from Eastern, Luapula and Southern respectively; one each from Northern and North-Western and none from Muchinga and Central provinces.

The ART status of the TB/HIV cases was not assessed in this study.

The majority of the TB/HIV cases had a positive culture result (94.4%; *p* = 0.000) and were found to have an abnormal chest x-ray (88.9%; *p* = 0.004) (Table 2). Also, half of the TB cases co-infected with HIV were smear positive (*p* = 0.000) and 52.8% had a cough lasting more than 2 weeks (*p* = 0.046). However, none of the 36 (100%) detected TB/HIV cases were on TB treatment at the time of the survey while 5/36 (13.9%) reported a previous history of TB treatment.

**Table 2:** Signs and symptoms, imaging results, bacteriology and treatment status among TB HIV con-infected cases as observed in the Zambia national TB prevalence survey; *N* = 36

	TB Cases co-infected with HIV *N* (%)	*p*-value
Cough ≥2 weeks	19 (52.8)	0.046
Fever ≥2 weeks	7 (19.4)	0.844
Chest pain ≥2 weeks	17 (47.2)	0.411
Chest x-ray abnormality	32 (88.9)	0.004
Smear positive	18 (50.0)	0.000
Culture positive	34 (94.4)	0.000
Currently on TB treatment	0 (0.0)	0.987
Ever treated for TB	5 (13.9)	0.646
Household member ever treated for TB	3 (8.3)	0.489

Table 3 highlights the signs, symptoms, imaging results, bacteriology and treatment status for TB cases with and those without or unknown HIV co-infection as observed in the Zambia national TB prevalence survey. Smear positivity and culture positivity predicted TB/HIV (*p* = 0.027 and *p* = 0.000) respectively. The HIV status was unknown for 43% of the observed survey cases. The signs, symptoms and bacteriology results among individuals found with TB in the survey, but who declined to be tested for HIV, were similar to those in whom an HIV test was done.

**Table 3:** Signs and symptoms, imaging results, bacteriology and treatment status for TB cases with and those without or unknown HIV co-infection as observed in the Zambia national TB prevalence survey

Variable	TB Cases with HIV N (%)	TB Cases without HIV N (%)	TB Cases with Unknown HIV status N (%)	Total N (%)
Number of cases	36 (13.6)	115 (43.4)	114 (43.0)	265 (100)
Cough ≥2 weeks	19 (52.8)	59 (51.3)	73 (64.0)	151 (57.0)
Fever ≥2 weeks	7 (19.4)	27 (23.5)	33 (28.9)	67 (25.3)
Chest pain ≥2 weeks	17 (47.2)	58 (50.4)	60 (52.6)	135 (50.9)
Chest x-ray abnormality	32 (88.9)	80 (43.5)	93 (81.6)	205 (77.4)
Smear positive	18 (50.0)	49 (42.6)	59 (51.7)	126 (47.5)
Culture positive	34 (94.4)	83 (72.2)	98 (86.0)	215 (81.1)
Currently on TB treatment	0 (0.0)	2 (1.7)	5 (4.4)	7 (2.6)
Ever treated for TB	5 (13.9)	13 (11.3)	17 (14.9)	35 (13.2)
Household member ever treated for TB	3 (8.3)	16 (13.9)	21 (18.4)	40 (15.1)

## Discussion

The prevalence of HIV among the TB cases tested in the survey was up to five times lower than routinely reported among clinical cases at facility level in the programmatic setting, where co-infection rates of 60–62% are reported [9, 15]. This implies that facility based data on co-infection rates alone may not reflect the true epidemiological picture of the general population. The difference is likely explained by two processes. On the one hand, TB-HIV co-infected patients are known to have poorer treatment outcomes [9] and therefore TB/HIV co-infected patients who do not seek care or cannot access care are likely to die early and do not remain as prevalent cases in the community. Furthermore, it is possible that TB/HIV co-infected individuals who fall sick are more likely to go to the health facility earlier because of the severity of symptoms and are thus potentially diagnosed earlier. It is also possible that the cases detected in this survey were in the early stage of disease since this was a TB screening of the general (healthy) population. In addition, with an active TB screening program among PLHIV for TB like in Zambia, it can be anticipated that those with known HIV are routinely screened for TB during ARV visits and hence TB was detected already and treated. Unfortunately in this survey it was not assessed whether persons already knew their HIV status or whether they were receiving ARV care. The implication for TB/HIV detection and management in a community setting require further investigation of the costs and benefits.

The TB/HIV co-infection rate among those tested was higher in the urban areas than in the rural settings; the geographical areas in which the prevalence of TB was higher, were also found to have higher levels of TB/HIV co-infections (as was the case for the highly urbanised Copperbelt and Lusaka Provinces of Zambia). This was similar to what has been reported from other countries such as China [16], where TB patients from HIV prevalent areas also reported higher HIV rates. The TB/HIV burden was also found to be higher within the age group that is also mainly affected by TB [9]. It thus appears that HIV may be a key driver of the TB epidemic in Zambia. This shows that although good progress has been made in implementing TB/HIV collaborative activities in Zambia as reported by Kapata and colleagues [9] there is need for sustaining effective coverage of interventions.

Notably, all the identified TB/HIV co-infected individuals were not on TB treatment at the time of the survey, similarly most (97%) of the TB cases without HIV were not anti-TB treatment. It should be noted that all individuals found with TB were included as cases irrespective of their treatment status and mycobacterial load. This could imply that these were HIV individuals in the early stage of TB and therefore had not yet sought care. This is more so as the screening procedures for the survey involved the application of chest X-rays and more sensitive diagnostic tools for diagnosis, namely culture methods and the Xpert MBT/RIF assay. There is need for implementation research to better understand the cost-effectiveness and health benefits of detecting TB/HIV early in the general population in resource constrained settings.

The majority of the identified TB/HIV cases had an abnormal chest x-ray while only 50% had a positive smear. This may imply that chest x-ray screening of HIV infected people for TB where possible may lead to increased TB case detection [17]. The role of chest x-rays in routine testing of people living with HIV should be explored further as part of the diagnostic algorithm for TB screening.

TB programs should continue to routinely screen for HIV in order to improve TB/HIV detection [18, 19]. On the other hand, HIV treatment centres should in fact scale up co-detection and co-management of TB as well so that TB/HIV co-infected individuals can be provided with comprehensive care and management [9]. Integration of TB/HIV diagnostic and treatment services in a one stop shop approach is key regardless of whether a patient entry into the health system is through the TB or HIV clinic, because they have mutual goals to find and treat cases [1, 9] however rigorous evaluation of the impact of the various models of integration are required [20]. Zambia should consider testing all presumptive TB cases for HIV in a bid to implement a more sensitive case detection algorithm.

The other important finding was that about a quarter of the TB/HIV co-infected patients who were detected in this study had a previous history of TB. The ART status of the participants was not assessed in this study unfortunately. However, it would be important to include ART status in future population level prevalence surveys so that the effect of ART on diseases duration could be understood in this context. In clinical settings in Zambia, TB/HIV collaborative activities have been improving with the proportion of TB cases tested for HIV rising from 23% in 2006, to 84% in 2010 but only 50% of the TB/HIV cases were put on ART [9]. Understanding the coverage of ART among TB/HIV cases can be useful in identifying areas of potential intervention so that the proportion of TB/HIV cases put on ART can be increased. Ensuring that ARVs are readily available in TB clinics may complement efforts to increase the number of TB/HIV patients on ART and lead to improved treatment outcomes of both diseases in the long term [21].

The national response to TB/HIV co-infection should be designed to pay particular attention to high burden areas when determining resource allocation. This will ensure that adequate resources are allocated to cause significant impact within reasonable time. Improving access to TB/HIV detection and management should be a priority on the national agenda; improvements in access to TB care have potential to yield positive outcomes [21]. One area of focus for intensive case detection could be the high-density urban areas where the prevalence of both HIV and TB is high [7]. Intensified TB case finding among people living with HIV is another area of TB/HIV response which could be further strengthened as a matter of priority.

The main limitation of this analysis is the high HIV testing refusal rate which could have introduced selection bias thereby affecting the precision of the reported estimates. The proportion of TB survey participants refusing HIV testing was 31.6% [14]; this potentially overestimated or underestimated the HIV-TB burden reported in this study depending on the characteristics of those who declined testing. If all decliners were HIV negative the prevalence would have been 14% (36/265) and if all were HIV positive, the prevalence would have been 57% (150/265). . Since the HIV testing refusal rates were higher among the younger survey participants, rural residents and the married [14] some bias may have been introduced in particular for these subgroups. Nonetheless, it remains key to address non acceptance of HIV testing in future surveys in order improve estimates and enhance appropriate care provision. Additionally, the survey was designed to detect pulmonary TB only hence there is a possibility of the occurrence of extra-pulmonary TB/HIV (EPTB/HIV), which was not investigated. This may have underestimated the true burden of TB/HIV in the general population, more so that HIV individuals tend to have EPTB as seen from a Zambian study [22] and routine surveillance data [23]. The use of *FASH* screening for extra-pulmonary TB (EPTB) detection may be considered in future surveys; *FASH* screening has been shown to detect EPTB in rural settings in Africa [24].

It is possible to use tuberculosis prevalence surveys to estimate the burden and characteristics of HIV among pulmonary TB patients specifically and in the general population. However, there is need for better yields in terms of the proportion of TB survey participants accepting an HIV test in order to reduce selection bias.

## Conclusions

The TB/HIV prevalence in the general population was found to be about five times lower than routinely reported among incident cases at facility level. TB/HIV co-infection rate was higher in areas with higher TB prevalence. Seeing that all TB/HIV co-infected individuals were not on anti-TB treatment; it is important to investigate innovative effective strategies for ensuring early case detection and treatment for these individuals. However, the costs and benefits of early detection and treatment of TB and HIV in a general population require further investigation in operational settings. Ensuring high acceptance of HIV testing in a community survey setting is key to improve estimates of TB/HIV co-infection.

## Abbreviations

ART: Antiretroviral therapy
EPTB: Extrapulmonary tuberculosis
HIV: Human immune virus
TB: Tuberculosis
